# *In vitro* activities of eravacycline against clinical bacterial isolates: a multicenter study in Guangdong, China

**DOI:** 10.3389/fmicb.2024.1504013

**Published:** 2024-11-18

**Authors:** Xiaoyan Liao, Qianwen Liang, Xinlu Dai, Shigang Wu, Chaohui Duan, Zhaofan Luo, Xiaoying Xie

**Affiliations:** ^1^Department of Clinical Laboratory, Sun Yat-sen Memorial Hospital, Sun Yat-sen University, Guangzhou, China; ^2^Department of Clinical Laboratory, The Seventh Affiliated Hospital, Sun Yat-sen University, Shenzhen, China; ^3^Department of Clinical Laboratory, Liwan Central Hospital of Guangzhou, Guangzhou, China

**Keywords:** eravacycline, MIC values, antimicrobial activity, common bacteria, tigecycline

## Abstract

**Introduction:**

Eravacycline (ERV), a novel tetracycline derivative, exhibits broad-spectrum antibacterial activity, but data on the bacterial activity against Chinese bacterial isolates are very scarce. This study aims to evaluate the activity of eravacycline against the common Gram-positive and Gram-negative bacteria isolates in Guangdong, China.

**Methods:**

The clinical isolates were collected from four centers between 1 November 2023 and 31 January 2024, and the susceptibility of eravacycline (MIC_50_, MIC_90_, and MIC) was determined using broth microdilution as a reference method and E-TEST strips to evaluate their consistency. A total of 594 strains were collected from the four centers, including *Staphylococcus aureus* (*n* = 126), *Enterococcus faecalis* (*n* = 58), *Enterococcus faecium* (*n* = 29), *Klebsiella pneumoniae* (*n* = 136), *Escherichia coli* (*n* = 187), and *Acinetobacter baumannii* (*n* = 58).

**Results and discussion:**

The MIC_50_ and MIC_90_ (mg/L) of eravacycline were 0.12 and 1 for S. aureus, 0.06 and 0.12 for *E. faecalis*, 0.06 and 0.5 for *E. faecium*, 0.25 and 0.5 for *E. coli*, 0.5 and 2 for *K. pneumoniae*, and 0.25 and 2 for *A. baumannii*. Based on the FDA and EUCAST breakpoints, the susceptibility of eravacycline against *S. aureus* was 46.03% vs. 83.33%, 56.90% vs. 94.93% against *E. faecalis*, and 62.07% vs. 79.31% in *E. faecium*. The susceptibility rates of *E. coli* and *K. pneumoniae* were 90.37% and 58.09, respectively. To evaluate the performance between the broth microdilution test (BMD) and ETEST methods, we compared essential agreement (EA), categorical agreement (CA), very major error (VME), and major error (ME). The results demonstrated that compared with BMD, eravacycline measured by ETEST had higher VME and ME referring to FDA breakpoints than EUCAST breakpoints in the Gram-positive isolates. Since there were no intermediate breakpoints for the eravacycline, the MIC values measured by the ETEST method might result in lower CA and higher VME and ME. This study provides MIC values of eravacycline against Gram-positive and Gram-negative pathogens in four hospitals in Guangdong Province, and eravacycline is an effective therapeutic candidate for common bacteria.

## Introduction

Eravacycline (ERV), a novel synthetic fluorocycline antibiotic, was approved by the US Food and Drug Administration (FDA) and the European Medicines Agency (EMA) in August 2018 for the treatment of complicated intra-abdominal infections (cIAIs) (Yusuf et al., 2021; Alosaimy et al., 2020). Compared to other tetracycline antibiotics, ERV is modified with a fluorine group at C7 position and a pyrrolidine group at C9 position (Lee and Burton, 2019), both of which contribute to the broad-spectrum antibacterial activity for the multi-antibiotics-resistant bacteria, including methicillin-resistant *Staphylococcus aureus* (MRSA) (Zhang et al., 2018; Monogue et al., 2016), vancomycin-resistant *enterococci* (VRE) (Tsai et al., 2021), and carbapenem-resistant *Enterobacterales* (CRE) (Yu et al., 2021; Chen et al., 2023; Koren et al., 2022; Johnston et al., 2020). In addition, eravacycline overcomes two tetracycline resistance mechanisms, including active efflux pumps and ribosomal protective proteins, making it more appealing to treating multidrug-resistant microorganisms. In brief, eravacycline combines with efflux pump TetM more closely than the other tetracycline and has a higher affinity for the ribosome and decreases *in vitro* translation, leading to higher drug concentrations than other drugs (Snydman et al., 2018; Hobbs et al., 2022).

On 16 March 2023, eravacycline was conditionally approved for market in China by the National Medical Products Administration (NMPA). On 27 July 2023, the first prescription was issued in Shanghai. Due to its short application time in China, there is a lack of sensitivity data on this new tetracycline derivative in China, especially in Guangdong, a South China province with a large population, high population mobility, developed economy, and particularly prominent problems of multidrug-resistant bacteria (data from the China Antimicrobial Resistance Surveillance System, CARSS).

The study aims to evaluate the *in vitro* activities of eravacycline against the main clinical bacterial strains, including *S. aureus, E. faecalis, E. faecium, E. coli, K. pneumoniae*, and *A. baumannii*, in four medical centers in Guangdong. The susceptibility of eravacycline is determined using broth microdilution as the reference method and E-TEST strips to evaluate their consistency, to supplement the lack of sensitivity data in China, provide data basis for clinical doctors' experience in medication, and provide reference for clinical laboratory testing.

## Materials and methods

### The flow chart of the study (Figure 1)

#### Participating institutions and ethical clearance

The study was conducted at four different teaching hospitals, two of which were at the South and North Hospitals of Sun Yat-Sen Memorial Hospital, and the other two were at Liwan Central Hospital of Guangzhou and the Seventh Affiliated Hospital of Sun Yat-Sen University. Each of the centers where the clinical strains were tested received approval or exemption from the local institutional review board before the study began.

**Figure 1:**
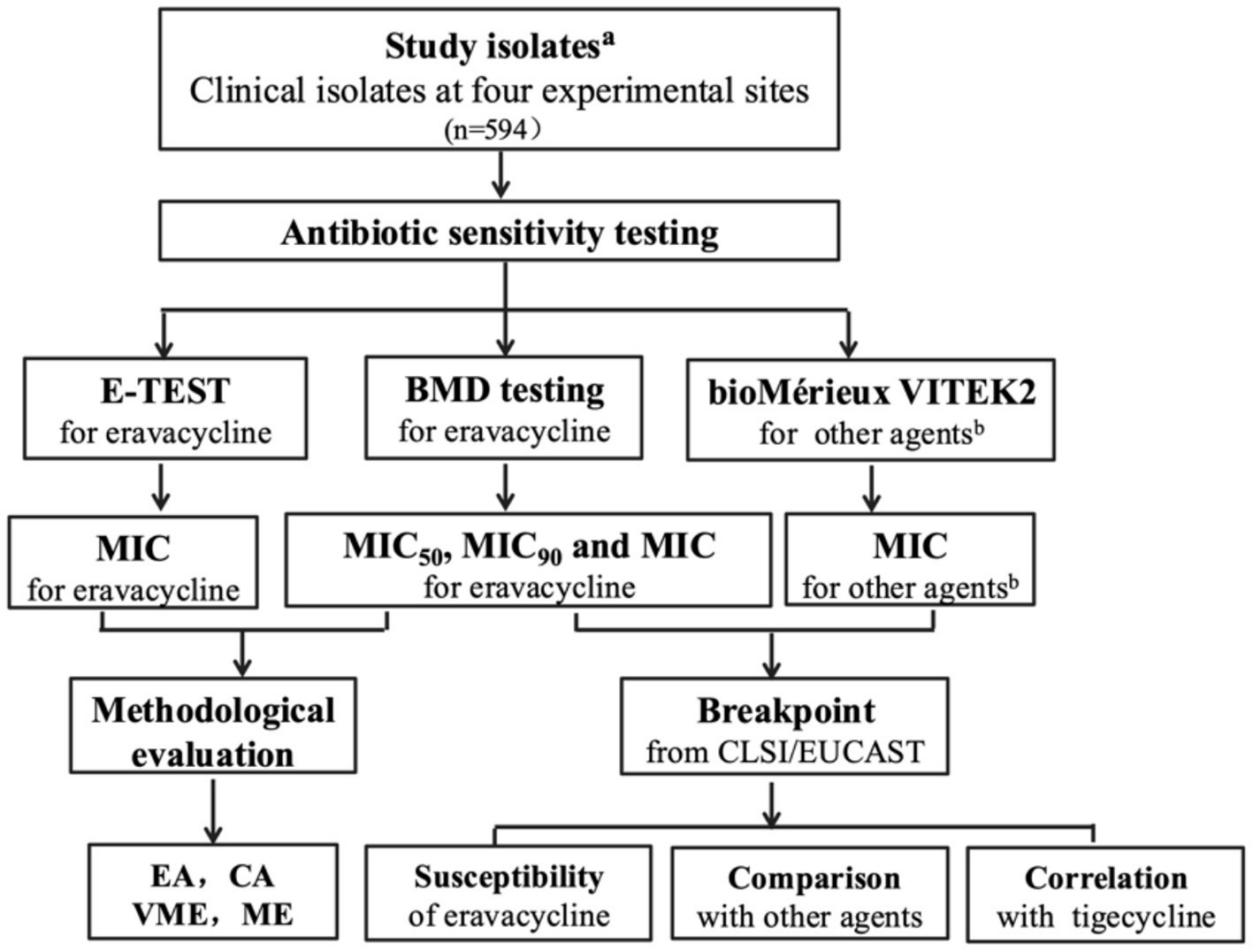
Comprehensive flow chart showing the objectives of different experiments and their mutual connectivity to the conclusion commemorates. ^a^Study isolates included *S. aureus, E. faecalis, E. faecium, K. pneumoniae, E. coli*, and *A. baumannii*; ^b^other agents included penicillin, oxacillin, ceftaroline, tigecycline, levofloxacin, moxifloxacin, erythromycin, clindamycin, gentamicin, trimethoprim–sulfamethoxazole, linezolid, teicoplanin, vancomycin, daptomycin, rifampicin, ampicillin, tetracycline, ciprofloxacin, nitrofurantoin, amoxicillin/clavulanic acid, piperacillin/tazobactam, cefoxitin, cefuroxime sodium, cefuroxime axetil, ceftriaxone, ceftazidime, cefepime, imipenem, ertapenem, amikacin, and cefoperazone sulbactam; BMD, broth microdilution; MIC, minimum inhibitory concentration; EA, essential agreement; CA, categorical agreement; VME, very major error; ME, major error.

#### Clinical bacterial isolates

From 1 November 2023 to 31 January 2024, clinical isolates (including 187 *E. coli*, 136 *K. pneumoniae*, 126 *S. aureus*, 58 *E. faecalis*, 29 *E. faecium*, and 58 *A. baumanii*) were collected from each center for a total of 594 strains. The isolates were obtained from different specimens, including sputum, blood, urine, bile, drain fluid, and skin pus. Matrix-assisted laser desorption ionization–time-of-flight mass spectrometry (MALDI-TOF MS) or Mérieux VITEK 2 Fully Automated Microbial Identification and Analysis System were used to identify the species of the clinical isolates. Clinical isolates were obtained from routine cultures processed in the clinical microbiology laboratory of each center. Screened clinical isolates were preserved in sheep blood and placed in a −80°C refrigerator. The technicians performing the clinical trial testing had no prior knowledge of the drug sensitivity results of any contemporary clinical isolates. Duplicate isolates from the same patient were excluded from the clinical trials.

#### Susceptibility testing methodology

The collected clinical isolates were transferred and grown on Columbia blood agar plates for 18–24 h. A single colony was taken from the blood agar plate, and a 0.5 MacFarland bacterial suspension was prepared using a turbidimeter in 0.85% saline. The prepared bacterial suspension was dipped in a sterile cotton swab within 15 min, spread evenly on the MH plate, and affixed to E-TEST strips. The MH plates were incubated in ambient air at 37°C, and the results were read after 18–24 h. The MIC between the two dilutions is rounded up to the next highest value. The broth microdilution method of drug sensitivity was carried out using 96-well plates according to the guidelines of the Clinical and Laboratory Standards Institute (CLSI M07-11th edition). The concentration gradients of eravacycline were 0.015, 0.03, 0.06, 0.12, 0.25, 0.5, 1, 2, 4, 8, and 16 mg/L. The treated 96-well broth microdilution plates were incubated at 37°C for 18–24 h. Minimum inhibitory concentration (MIC) was defined as the absence of turbidity observed by the naked eye against a black background. The results of this study were interpreted according to the standards of the European Committee on Antimicrobial Susceptibility Testing (2021) and the standards of the US Food and Drug Administration (FDA, 2020).

#### QC study

Three ERV E-TEST ATCC reference strains and four BMD ATCC reference strains, as recommended by CLSI M100 and EUCAST, were tested as QC on each day of the quality control. The QC strains included *Escherichia coli* ATCC25922 (CLSI/EUCAST range, 0.032 to 0.125 mg/L), *Enterococcus faecalis* ATCC29212 (CLSI/EUCAST range, 0.016 to 0.064 mg/L), and *Pseudomonas aeruginosa* ATCC27853 (CLSI range, 2 to 16 mg/L). *Staphylococcus aureus* ATCC29213 was used solely for BMD (CLSI/EUCAST range, 0.016 to 0.125 mg/L). QC strains were passaged twice before testing. If the QC results were out of range, the results were considered invalid.

#### Data analysis

A two-sample proportionality test was used to compare the drug sensitivity rates of different antibiotics. A two-sided *P* < 0.05 was considered statistically significant. Spearman's correlation coefficient was used to analyze the correlation between MICs of different antibiotics. We defined rho >0.7 as a strong correlation, rho 0.40–0.69 as a moderate correlation, and 0 < rho < 0.39 as a weak correlation (Schober et al., 2018). Data were analyzed by SPSS software (version 29.0.1.0).

## Results

### The percentage of susceptibility and the cumulative percentage of MIC values of eravacycline

In this study, from November 2023 to January 2024, a total of 594 strains were collected from the South and North Hospitals of Sun Yat-Sen Memorial Hospital, and the other two were at Liwan Central Hospital of Guangzhou and the Seventh Affiliated Hospital of Sun Yat-Sen University. The isolates were obtained from different specimens, including sputum, blood, urine, bile, drain fluid, and skin pus. *E. coli* (*n* = 187) was the most abundant genus, followed by *K. pneumoniae* (*n* = 136), *S. aureus* (*n* = 126), *A. baumannii* (*n* = 58), *E. faecalis* (*n* = 58), and *E. faecium* (*n* = 29). The eravacycline MIC values were determined by broth microdilution. In Table 1, the MIC_50_ and MIC_90_(mg/L) of eravacycline were 0.25 and 0.5 for *E. coli*, 0.5 and 2 for *K. pneumoniae*, 0.12 and 1 for *S. aureus*, 0.5 and 2 for *A. baumannii*, 0.06 and 0.12 for *E. faecalis*, and 0.06 and 0.5 for *E. faecium*. The distribution of eravacycline MIC for Gram-positive and Gram-negative isolates is shown in Supplementary Table 1 in detail.

**Table 1:** *In vitro* the percentage of susceptibility to eravacycline against gram-positive cocci and gram-negative bacilli.

**Organism**	**N**	**MIC_50_ (mg/L)**	**MIC_90_ (mg/L)**	**MIC range (mg/L)**	**Susceptibility %**	
					**FDA breakpoints**	**EUCAST breakpoints**
*S. aureus*	126	0.12	1	0.03–1	46.03	83.33
*E. faecalis*	58	0.06	0.12	0.015–1	56.90	94.93
*E. faecium*	29	0.06	0.5	0.03–4	62.07	79.31
*E. coli*	187	0.25	0.5	0.015–8	90.37	90.37
*K. pneumoniae*	136	0.5	2	0.06–8	58.09	NA
*A. baumannii*	58	0.25	2	0.03–4	NA	NA

MIC, minimum inhibitory concentration; NA, not applicable; N, numbers.

When using EUCAST breakpoints, the susceptibility to eravacycline against *Staphylococcus aureus* was 83.33%; however, the FDA breakpoints set two dilutions lower than EUCAST (≤ 0.06 mg/L vs. ≤ 0.25 mg/L), resulting in a reduced susceptibility to 46.03%. The breakpoint effect can also be observed in *Enterococci*, where the FDA breakpoints set one dilution lower than EUCAST (≤ 0.06 mg/L vs. ≤ 0.12 mg/L); 94.93% of *E. faecalis* isolates were susceptible to eravacycline, whereas the rate dropped to 56.90% when applying the FDA breakpoints (≤ 0.06 mg/L). The ERV susceptibility of *E. faecium* was 79.31% by EUCAST but only 62.07% by FDA. It is worth noting that the FDA and EUCAST suggest the same breakpoints for ERV against *E. coli*. With the FDA and EUCAST breakpoints, *E. coli* was 90.37% susceptible to eravacyline, higher than *K. pneumoniae* isolates (58.09%), according to the FDA breakpoints. As there is no information about setting EUCAST breakpoints for *K. pneumoniae* and clinical breakpoints against *A. baumannii*, neither the FDA nor the EUCAST, we could not determine which strain was susceptible or resistant. However, the low MIC values (< 4 mg/L) demonstrated eravacycline was effective against *A. baumannii*.

### The correlation of eravacycline and tigecycline

**Figure 2:**
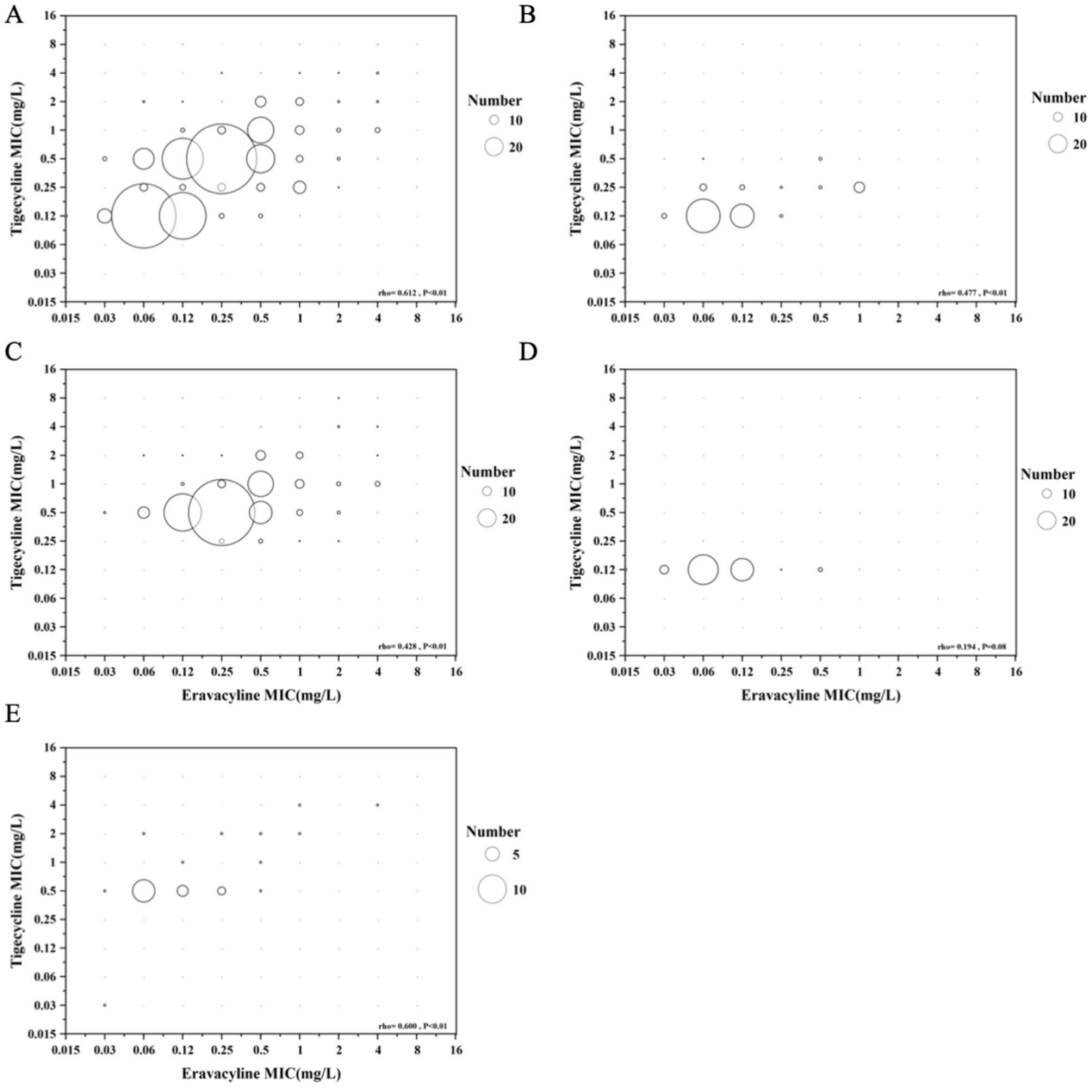
Bubble plots compare the correlation between eravacycline and tigecycline against all collected isolates **(A)**, *S. aureus* **(B)**, *Enterobacteriaceae* **(C)**, *Enterococcus spp* **(D)**, and *A. baumannii* **(E)**. The bubble sizes indicate the isolate amounts.

To compare the correlation between the eravacycline and tigecycline (Figure 2), the eravacycline MIC values were evaluated by broth microdilution, and the tigecycline MIC values were determined by VITEK 2. In a total of 508 isolates, the MIC distribution results were moderately correlated between eravacycline and tigecycline (rho = 0.612, *P* < 0.01). Of 115 *S. aureus*, the MIC distribution results were also moderately correlated between eravacycline and tigecycline (rho = 0.477, *P* < 0.01), and the MIC distribution results of *Enterobacteriaceae*, including *E. coli* and *K. pneumoniae*, correlated between eravacycline and tigecycline (rho = 0.428, *P* < 0.01). Of the 83 *Enterococcus spp*, there was no difference in correlation between eravacycline and tigecycline (rho = 0.194, *P* = 0.08). The MIC results of 25 *A. baumannii* were also moderately correlated with eravacycline and tigecycline (rho = 0.600, *P* < 0.01). In comparison with the MIC values, the MIC results with tigecycline were higher than eravacycline in most situations.

### *In vitro* susceptibility of eravacycline-resistant strains (according to FDA breakpoints) and comparator antibiotics

To evaluate the eravacycline-resistant strains and comparator antibiotics, the MIC values of eravacycline were determined by broth microdilution, and the other antibiotics were determined by VITEK 2 (Table 2). Based on FDA breakpoints, of the 67 eravacycline-resistant *S. aureus*, 100% were sensitive to ceftaroline, tigecycline, linezolid, teicoplanin, vancomycin, and daptomycin, and approximately 40% were sensitive to oxacillin, levofloxacin, moxifloxacin, erythromycin, and clindamycin. Only 13.43% were sensitive to penicillin. Of 24 eravacycline-resistant *E. faecalis*, 100% were sensitive to penicillin, ampicillin, tigecycline, and vancomycin, 95% to nitrofurantoin, only 5% to tetracycline, and 4.17% to erythromycin. Of 11 eravacycline-resistant *E. faecium*, 100% were sensitive to linezolid, 90.09% to vancomycin, 72.72% to tigecycline, 9.09% to penicillin, and 100% to ampicillin and ciprofloxacin. Among the *Enterobacteriaceae*, of the 18 eravacycline-resistant *E. coli*, 100% were sensitive to tigecycline, 84.61% to cefoperazone sulbactam, and only 8.33% to cefuroxime axetil. Similar to *Escherichia coli*, of the 56 eravacycline-resistant *K. pneumoniae*, 75.61% were sensitive to tigecycline, and < 10% were sensitive to cefuroxime axetil, cefuroxime sodium, and levofloxacin.

**Table 2:** *In vitro* susceptibility of eravacycline-resistant strains (according to FDA breakpoints) to comparator antibiotics.

**Organism (*n*)**	**Antimicrobial agents**	**MIC(mg/L)**		**MIC range (mg/L)**	**Susceptibility %**
		**MIC50**	**MIC90**		
*S. aureu* (*n* = 67)	Penicillin	0.5	2	0.06–2	13.43%
	Oxacillin	4	4	0.25–4	47.05%
	Ceftaroline	0.25	1	0.06–1	100.00%
	Tigecycline	0.25	0.5	0.12–0.5	100.00%
	Levofloxacin	0.5	8	0.12–8	47.05%
	Moxifloxacin	0.25	2	0.25–8	45.58%
	Erythromycin	8	8	0.25–8	43.28%
	Clindamycin	4	4	0.25–4	43.28%
	Gentamicin	1	4	0.5–16	69.11%
	Trimethoprimsulfamethoxazole	0.5	4	0.5–4	97.05%
	Linezolid	1	2	1–2	100.00%
	Teicoplanin	1	2	0.5–4	100.00%
	Vancomycin	1	1	0.5–1	100.00%
	Daptomycin	0.25	0.5	0.12–0.5	100.00%
	Rifampicin	0.5	0.5	0.5–4	97.05%
*E. faecalis* (*n* = 24)	Penicillin	4	8	1–8	100.00%
	Ampicillin	2	2	0.5–2	100.00%
	Tetracycline	16	16	1–16	5.00%
	Tigecycline	0.12	0.12	0.12–0.25	100.00%
	Ciprofloxacin	1	8	0.5–8	55.00%
	Levofloxacin	1	8	0.5–8	55.00%
	Erythromycin	8	8	0.25–8	4.17%
	Linezolid	2	8	1–8	78.26%
	Vancomycin	1	2	0.5–2	100.00%
	Nitrofurantoin	16	16	16–64	95.00%
*E. faecium* (*n* = 11)	Penicillin	8	64	8–64	9.09%
	Ampicillin	32	32	4–32	0.00%
	Tetracycline	16	16	1–16	10.00%
	Tigecycline	0.12	0.5	0.12–2	72.72%
	Ciprofloxacin	8	8	2–8	0.00%
	Levofloxacin	8	8	2–8	20.00%
	Erythromycin	8	8	0.25–8	9.09%
	Linezolid	2	2	1–2	100.00%
	Vancomycin	0.5	1	0.5–32	90.90%
	Nitrofurantoin	32	128	32–256	50.00%
*E. coli* (*n* = 18)	Amoxycillin/clavulanic acid	8	32	2–32	52.94%
	Piperacillin/tazobactam	8	128	4–128	61.11%
	Cefoxitin	8	32	4–64	55.55%
	Cefuroxime sodium	64	64	16–64	15.38%
	Cefuroxime axetil	64	64	16–64	8.33%
	Ceftriaxone	64	64	0.25–64	46.15%
	Ceftazidime	8	32	0.12–64	44.44%
	Cefepime	8	16	0.12–16	44.44%
	Imipenem	0.25	8	0.25–16	77.78%
	Ertapenem	0.12	4	0.12–8	72.22%
	Tigecycline	0.5	2	0.25–2	100.00%
	Levofloxacin	8	8	0.12–8	11.11%
	Amikacin	2	64	2–64	77.78%
	Cefoperazone sulbactam	8	64	8–128	84.61%
	Trimethoprim–sulfamethoxazole	4	320	1–320	44.44%
*K. pneumoniae* (*n* = 56)	Trimethoprim–sulfamethoxazole	4	320	1–320	10.71%
	Cefoperazone/sulbactam	16	64	8–64	37.50%
	Amikacin	4	64	2–64	57.14%
	Levofloxacin	8	8	0.12–8	8.93%
	Ertapenem	4	64	0.12–64	34.54%
	Imipenem	8	16	0.25–16	41.07%
	Cefepime	16	32	0.12–32	21.43%
	Ceftazidime	16	64	0.5–64	21.42%
	Ceftriaxone	4	64	0.25–64	23.63%
	Cefuroxime Axetil	64	64	2–64	7.27%
	Cefuroxime Sodium	64	128	2–128	7.84%
	Cefoxitin	32	64	4–64	27.27%
	Piperacillin/tazobactam	128	128	2–128	23.21%
	Amoxycillin/clavulanic acid	32	32	2–32	27.27%
	Tigecycline	1	4	0.25–8	75.61%

N, numbers; MIC, minimum inhibitory concentration.

### Eravacycline performance with FDA breakpoints and EUCAST breakpoints against gram-positive and gram-negative isolates

To evaluate eravacycline performance, eravacycline MIC values were measured by the broth microdilution method and ETEST strips. According to FDA breakpoints, of 126 *S. aureus* were 91.26% (115/126 isolates) EA, 92.06% (116/126 isolates) CA, VME rate of 22.22% (28/126 isolates), ME rate of 2.38% (3/126 isolates), 18 of VMEs, and 3 of MEs were within EA (Table 3). Dated back to 3 November 2015, according to the FDA response to the STMA letter, when the VME or ME was not acceptable for the antibiotic in which there is no intermediate point, the VME rate was adjusted to exclude the VME within EA (Blanchard et al., 2023). The adjusted VME rate was 7.93% (10/126 isolates). Of 58 *E. faecalis*, EA was 86.20% (50/58 isolates), CA was 86.21% (50/58 isolates), the adjusted VME was 12.07% (7/58 isolates), and ME was 1.72% (1/58 isolates). Of the 29 *E. faecium*, CA was 93.10% (27/29), 2 VMEs were within EA, and adjusted VME was 6.89% (2/29 isolates). With the EUCAST breakpoints, among *S. aureus*, the CA rate was 97.61% (123/126 isolates), and VME rate was 2.38% (3/126). Among *E. faecium*, CA was 89.66% (26/29) and VME was 10.34% (3/29 isolates) (Table 4). Among *E. coli*, EA was 88.23% (165/187 isolates), CA was 91.97% (172/187 isolates), and adjusted VME was 6.42% (12/187 isolates) (Table 3). The breakpoint of *K. pneumoniae* was suggested by the FDA, and EA was 91.27% (124/136 isolates), CA was 95.59% (130/136 isolates), adjusted VME was 3.68% (5/136 isolates), and ME was 0.74% (1/136). Of 58 *A. baumannii*, because there are no breakpoints suggested by FDA or EUCAST, CA, VME, and ME could not be analyzed and the EA was 86.20% (50/58 isolates). The FDA and ISO performance criteria were used to evaluate the performance, as follows: EA and CA (≥90%), ME rate (≤ 3%), VME rate (≤ 2%) (FDA), or (≤ 3%) (ISO) [FDA 2009, ISO 20776-2:2021] (Food Drug Administration, 2009; International Standards Organization, 2021). The ETEST ERV did not meet the criterion for the clinical strains.

**Table 3:** Eravacycline performance with FDA breakpoints.

**Organism**	**Total no**.	**No. within EA**	**EA(%)**	**No. within CA**	**CA(%)**	**No. of VME**	**No. of MEs**
*S.aureus*	126	115	91.26	116	92.06	10	0
*E. faecalis*	58	50	86.20	50	86.21	7	1
*E. faecium*	29	26	89.60	27	93.10	2	0
*E. coli*	187	165	88.23	175	93.58	12	0
*K. pneumoniae*	136	124	91.27	130	95.59	5	1
*A. baumannii*	58	50	86.20	NA	NA	NA	NA

No, numbers; NA, not applicable; EA, essential agreement; CA, categorical agreement; VME, very major error; ME, major error.

**Table 4:** Eravacycline performance with EUCAST breakpoints.

**Organism**	**Total no**.	**No. within EA**	**EA(%)**	**No. within CA**	**CA (%)**	**No. of VME**	**No. of MEs**
*S.aureus*	126	115	91.26	123	97.61	3	0
*E. faecalis*	58	50	86.20	54	93.10	3	1
*E. faecium*	29	26	89.60	26	89.66	3	0
*E. coli*	187	165	88.23	172	91.97	15	0
*K. pneumoniae*	136	124	91.27	NA	NA	NA	NA
*A. baumannii*	58	50	86.20	NA	NA	NA	NA

No, numbers; NA, not applicable; EA, essential agreement; CA, categorical agreement; VME, very major error; ME, major error.

## Discussion

Eravacycline (ERV), as a new tetracycline derivative, has been approved for complex abdominal infections and exhibits broad-spectrum antibacterial activity similar to the other family member, tigecycline. The spectrum covers *Staphylococcus spp, Enterococcus spp, Enterobacteriaceae*, and anaerobic microorganisms (Karvouniaris et al., 2023; Bassères et al., 2020). In addition, eravacycline also provides an alternative to treating infections caused by difficult-to-treat organisms, including MRSA and VRE, as well as many GNB, including *Enterobacteriaceae* resistant to carbapenems, cephalosporins, fluoroquinolones, and β-lactam/β-lactamase inhibitor combinations (Zhang et al., 2016; Brauncajs et al., 2023; Rolston et al., 2023; Bonnin et al., 2023).

In this study, we evaluated the eravacycline *in vitro* against 594 bacterial strains, including *S. aureus, E. faecalis, E. faecium, E. coli, K. pneumoniae, and A. baumannii*, in four hospitals in Guangdong Province. In addition, we compared the correlation between eravacycline and tigecycline, figured out the susceptibility of eravacycline-resistant strains and comparator antibiotics, and evaluated the performance of the microbroth dilution reference method and ERV ETEST method according to FDA and EUCAST breakpoints. MIC values of Gram-positive bacteria were lower than Gram-negative isolates. Following the EUCAST breakpoints, ERV susceptibility was higher than using the FDA breakpoints: 83.33% vs. 46.03% in *S. aureus*, 94.93% vs. 56.90% in *E. faecalis*, and 79.31% vs. 62.07% in *E. faecium* (Table 1). Therefore, it is important to re-evaluate ERV breakpoints to harmonize the two breakpoints; otherwise, there is a risk of inappropriate treatment. *E. coli* was 90.37% susceptible to ERV, whereas the susceptibility rate of *K. pneumoniae* was only 58.09%, which was different from Hawser S.'s study (Hawser et al., 2023) and similar to Huang's study in Taiwan and Zou's study in Zhejiang. That may be explained by different study settings and environmental differences (Huang et al., 2023; Zou et al., 2023; Galani et al., 2023). The MIC values of *K. pneumoniae* in China were higher than those in Europe, the United States, and Canada (Zhanel et al., 2018; Zheng et al., 2018). Moreover, resistance to eravacycline may be due to efflux pump, drug resistance gene mutations involving *ramR* and *rpsJ*, and heteroresistance of ERV in clinical isolates (Galani et al., 2023; Abdallah et al., 2015; Zeng et al., 2022; Wen et al., 2020). Since there were no breakpoints for *A. baumannii*, the susceptibility of ERV could not be figured out, but MIC_90_ was 2 mg/L, which is similar to the study conducted by Deolankar et al. (2022). Therefore, eravacycline is still effective for the treatment of *A. baumannii*. We also found that ERV had higher antibacterial activity against common bacteria compared with antibiotic multi-resistance bacteria, such as MRSA and CRE (Supplementary Table 2). Since there were limited numbers of *Enterococcus spp*, we could not figure out the precise sensitivity of the *Enterococcus spp*, which was a limitation of the study.

We performed Spearman's correlation tests for MIC values between the eravacycline and tigecycline (Figure 2). The MIC results of two antibiotics were moderately correlated in *S. aureus* (rho = 0.477), *A. baumannii* (rho = 0.600), Enterobacteriaceae (rho = 0.428), and the selected isolates (rho = 0.612). There was no correlation in Enterococcus spp. (rho = 0.194). The results implied that eravacycline and tigecycline were similar, because both of them were tetracycline derivatives. Further clinical studies are needed to apply these findings to clinical practice *in vitro*.

Based on FDA breakpoints (Table 2), to evaluate eravacycline-resistant strains to the other antibacterial agents, the results showed that among the eravacycline-resistant *S. aureus*, the susceptibility of ceftaroline, tigecycline, linezolid, teicoplanin, vancomycin, and daptomycin was high but penicillin was less sensitive than those (only 13.43%). Of the eravacycline-resistant *E. faecalis*, most of them were still sensitive to penicillin, ampicillin, tigecycline, vancomycin, and nitrofurantoin, whereas the majority of the eravacycline-resistant *E. faecium* were resistant to penicillin and ampicillin, but most of them were sensitive to linezolid and vancomycin. The susceptibility of penicillin and ampicillin against *E. faecalis* was higher than those among *E. faecium*, and that is why the results show a great difference between the eravacycline-resistant *E. faecalis* and *E. faecium* (Morrissey et al., 2020a). In addition, vancomycin and linezolid were effective *in vitro* for ERV-resistant Gram-positive bacterial isolates. Tigecycline still maintains an antibacterial effect on some ERV-resistant Gram-negative isolates. Considering the numbers of ERV-resistant bacterial isolates were limited, we need more ERV-resistant isolates for further study.

Among the Gram-positive bacteria, the susceptibility of tigecycline and vancomycin against eravacycline-resistant isolates was high. Of the eravacycline-resistant *Enterobacteriaceae* including *E. coli* and *K. pneumoniae*, both of them showed high susceptibility to tigecycline, which were similar to Lutgring, J. D., in the United States (Lutgring et al., 2020), but were contrary to the conclusions in the research written by Huang C.F. and Morrissey I (Huang et al., 2023; Morrissey et al., 2020b). In this study, we found that eravacycline had lower MIC values but tigecycline had better antibacterial activity, as the breakpoints were different. The FDA and EUCAST breakpoints suggest lower breakpoints for the eravacycline than the tigecycline. Notably, EUCAST and FDA suggest the same susceptible breakpoint for tigecycline among the *Staphylococci* (≤ 0.5 mg/L) and *Enterococci* (≤ 0.25 mg/L) but were different in *Enterobacteriaceae* (≤ 0.5 mg/L vs. ≤ 2 mg/L). Considering both eravacycline and tigecycline susceptible breakpoints published by the EUCAST were ≤ 0.5 mg/L. The tigecycline-susceptible breakpoint by the FDA was considered to be too high, as previously reported (Hawser et al., 2023).

We compared the ERV ETEST strips and reference method to evaluate the performance (Tables 3, 4). Contrary to the results evaluated by Blanchard et al. (2023), among the Gram-positive bacteria, since the EUCAST breakpoints are higher than the FDA, the lack of the intermediate interpretive category might lead to the results with the *S. aureus, E. faecium*, and *E. faecalis* having more potential for VMEs or MEs compared with broth microdilution. The susceptible breakpoints suggested by FDA and EUCAST were the same (≤ 0.5 mg/L). Before adjusting the VMEs and MEs, there were no differences among *Enterobacteriaceae*. It is noteworthy that the latest product, VITEK 2 AST-XN18, researched by bioMérieux has been on the market recently and offers an alternative method to evaluate the susceptibility of ERV *in vitro*.

In the current study, there were still some limitations. First, the results presented in this study did not represent the whole population, and more research was needed to fully understand the antibacterial activity of eravacycline. Second, although the eravacycline was designed for the treatment of complicated intraabdominal infections, the isolates we collected still included the blood and urine samples. Third, the mechanisms of eravacycline-resistant clinical isolates have not been discussed in detail. Our further study is going to evaluate other tetracyclines such as omadacycline, oxymycin, minomycin, and doxymycin. In addition, more mechanism research studies on the ERV resistance isolates will be carried out and explore the combined effects of ERV with other antibiotics.

Overall, eravacycline, a novel tetracycline derivative, has lower MIC_50_ and MIC_90_ values against most common bacteria compared with tigecycline. It is urgent to optimize antimicrobial management while using new antimicrobial agents. It is of great significance to evaluate the susceptibility of these new drugs *in vitro*.

## Data Availability

The original contributions presented in the study are included in the article/Supplementary material, further inquiries can be directed to the corresponding authors.
