# Supplementary material for: In vitro activities of eravacycline against clinical bacterial isolates: a multicenter study in Guangdong, China
Source: Front Microbiol. 2024 Nov 18;15:1504013. doi: 10.3389/fmicb.2024.1504013 (PMC11609169; doi:10.3389/fmicb.2024.1504013)
Supplement: Supplementary file 1 [file Data_Sheet_1.pdf]

Supplementary materials

Table 1 Cumulative percent distribution of eravacycline MIC against Gram-positive and Gram-negative isolates.

		Cumulative % of isolates with the following eravacycline MIC(mg/L)										
organism	N	0.015	0.03	0.06	0.12	0.25	0.5	1	2	4	8	16
S.aureus	126	0.00	6.35	46.03	76.98	83.33	89.68	100.00	100.00	100.00	100.00	100.00
E. faecalis	58	6.90	12.07	56.90	94.83	94.83	98.28	100.00	100.00	100.00	100.00	100.00
E.faecium	29	0.00	27.59	62.07	79.31	86.21	96.55	96.55	96.55	100.00	100.00	100.00
E.coli	187	0.53	2.14	9.09	33.69	75.40	90.37	97.86	98.93	99.47	100.00	100.00
K.pneumoniae	136	0.00	0.00	1.47	6.62	19.85	58.09	75.74	90.44	99.26	100.00	100.00
A.baumannii	58	0.00	1.72	29.31	41.38	51.72	70.69	87.93	93.10	100.00	100.00	100.00

N : Numbers

Table 1 shows the distribution of eravacycline MIC for gram-positive and gram-negative isolates. The MIC range of eravacycline were 0.03-1 mg/L for *Staphylococcus aureus*, 0.015-1 mg/L for *Enterococcus faecalis*, 0.03-4 mg/L for *Enterococcus faecium*, 0.015-8 mg/L for *Escherichia coli*, 0.06-8mg/L for *Klebsiella pneumoniae*, and 0.03-4 mg/L for *Acinetobacter baumannii*. The gram-positive bacteria were more susceptible to eravacycline than the gram-negative isolates with MIC_{90} values of 0.12–1 mg/L for the Gram-positive isolates and 0.5-2 mg/L for the Gram-negative strains.

TABLE 2 In vitro the percentage of susceptibility to eravacycline against Gram-positive cocci and Gram-negative bacilli

organism	N	MIC_{50} (mg/L)	MIC_{90} (mg/L)	MIC Range (mg/L)	Susceptibility % FDA breakpoints	Susceptibility % EUCAST breakpoints
MRSA(*S. aureus*)	35	0.12	1	0.06-1	45.71	68.75
VRE(*E. faecalis*)	0	-	-	-	-	-

VRE (*E. faecium*)	2	0.03	0.06	0.03-0.06	100	100
CRE(*E. coli*)	17	0.25	8	0.25-8	76.47	76.47
CRE(*K. pneumoniae*)	35	2	4	0.5-8	11.43	NA
CR(A. *baumannii*)	30	1	2	0.03-4	NA	NA

N: Numbers

MRSA: Methicillin-resistant *S.aureus*

VRE: Vancomycin-resistant *Enterococcus*

CRE: Carbapenem-resistant *Enterobacteriaceae*

CR: Carbapenem-resistant

To evaluate the ERV against drug-resistant bacteria in vitro, methicillin-resistant *Staphylococcus aureus* (MRSA), vancomycin-resistant *E. faecalis and E. faecium,* carbapenem-resistant *E.coli* and *K. pneumoniae,* carbapenem-resistant *A. baumannii* were measured by broth microdilution. Of 45 MRSA, only 45.71% and 68.75% were sensitive to ERV based on the FDA and EUCAST breakpoints, which was lower than the *S. aureus*. However, only limited numbers of the VRE, we couldn't not figure out the susceptibility against the vancomycin-resistant *E. faecalis* and *E. faecium.* Of carbapenem-resistant *E. coli*, 76.47% sensitive to ERV but the carbapenem-resistant *K. pneumoniae,* only 11.43% sensitive to ERV. Of 30 carbapenem-resistant *A. baumannii*, MIC_{50} was 1mg/L, which was lower than *A.baumannii*.
